# Use of an Interactive Obesity Treatment Approach in Individuals With Severe Mental Illness: Feasibility, Acceptability, and Proposed Engagement Criteria

**DOI:** 10.2196/38496

**Published:** 2022-12-13

**Authors:** Ginger Nicol, Madeline Jansen, Rita Haddad, Amanda Ricchio, Michael D Yingling, Julia A Schweiger, Katie Keenoy, Bradley A Evanoff, John W Newcomer

**Affiliations:** 1 Department of Psychiatry Washington University School of Medicine St. Louis, MO United States; 2 Division of Child and Adolescent Psychiatry Department of Psychiatry Los Angeles David Geffen School of Medicine, University of California Los Angeles, CA United States; 3 Department of Psychiatry Washington University School of Medicine St Louis, MO United States; 4 Washington University School of Medicine St. Louis, MO United States; 5 Division of General Medical Sciences Department of Internal Medicine Washington University School of Medicine St. Louis, MO United States

**Keywords:** obesity, mentally ill people/persons, health services, mobile health

## Abstract

**Background:**

Digital and mobile health interventions are increasingly being used to support healthy lifestyle change, including in certain high-risk populations such as those with severe mental illnesses (SMIs). Life expectancy in this population lags 15 years behind counterparts in the general population, primarily due to obesity-related health conditions.

**Objective:**

We tested the feasibility and usability of a 12-week interactive obesity treatment approach (iOTA) to adults with chronic SMIs (depression, bipolar disorder and schizophrenia spectrum disorder) receiving treatment in community settings. The iOTA incorporates short message service (SMS) text messages to supplement monthly in-person health coaching.

**Methods:**

Factors hypothesized to be associated with weight change were illness severity and treatment engagement. Severe psychiatric symptoms were defined as baseline Clinical Global Impression severity score of >5. Criterion engagement was defined as a text messaging response rate >80% during the first 4 weeks of treatment. Disordered eating, assessed with the Loss of Control Over Eating Scores, was also evaluated. Participants provided qualitative data, further informing assessment of intervention feasibility, usability, and acceptability.

**Results:**

A total of 26 participants were enrolled. The mean age was 48.5 (SD 15.67) years; 40% (10/26) were Black and 60% (15/26) female. Participants with lower symptom severity and adequate engagement demonstrated significantly decreased weight (*F*_1,16_=22.54, *P*<.001). Conversely, high symptom severity and lower text message response rates were associated with trend-level increases in weight (*F*_1,7_=4.33, *P*=.08). Loss-of-control eating was not observed to impact treatment outcome. Participants voiced preference for combination of live health coaching and text messaging, expressing desire for personalized message content.

**Conclusions:**

These results demonstrate the feasibility of delivering an adapted iOTA to SMI patients receiving care in community settings and suggest testable criteria for defining sufficient treatment engagement and psychiatric symptom severity, two factors known to impact weight loss outcomes. These important findings suggest specific adaptations may be needed for optimal treatment outcomes in individuals with SMI.

## Introduction

The field of obesity medicine has embraced the concept that excess adiposity is a disease state with important neurobehavioral causes and consequences [1-3]. Thus, sustained weight management may not be achievable by altering energy balance so that the amount of energy consumed (eg, calories) is less than the amount of energy expended (eg, during exercise), as evidenced by emerging treatments that target mechanisms involving central reward and peripheral modulation of satiety neurocircuitry [4], insulin sensitivity [5,6], and inflammation [7]. Nonetheless, the primary tenets of successful weight loss remain deeply rooted in lifestyle change, with interventions including intensive coaching interactions, highly trained interventionists, and robust clinical infrastructure being most effective [8]. Behavioral phenotyping of individuals achieving long-term weight control suggests that the ability to sustain negative energy balance with a combination of dietary restriction and high levels of physical activity, along with frequent self-monitoring, are indicators of long-term success [9]. However, individuals who sustain clinically significant weight loss also have greater distress over body image and are more likely to engage in disordered eating behaviors [10]. Psychiatric comorbidity is common in treatment-seeking populations [11,12], with eating disorders being more prevalent in high-weight individuals than in those with normal or low weight [13]. These complicating factors contribute to delayed or suboptimal treatment engagement and response [14-16].

Patients with chronic severe mental illness (SMI) are at high risk for developing obesity and related adverse health conditions, attributable to treatment with obesogenic medications [17], sedentary lifestyle [18], and unbalanced dietary intake [19]. Individuals with SMI express a preference for mobile access to behavioral treatments [20,21] but report unique challenges to engagement in lifestyle changes that are specific to the type and severity of their behavioral health symptoms [22,23]. Behavioral interventions to reverse preexisting obesity in chronic SMI demonstrate modest effectiveness during active intervention [22,24,25], but longer term benefits are attenuated or reduced, in part due to limited engagement [16,26-28]. As in the general population, more frequent contacts and longer intervention periods are associated with better adherence and long-term effectiveness [29,30]. However, staffing and other cost requirements (eg, gym memberships) may impact large-scale implementation efforts [22,25,31-33]. Incorporation of mobile health (mHealth) components into lifestyle interventions specifically adapted for people with SMIs treated in community settings may improve scalability, engagement and long-term maintenance effects.

Interactive obesity treatment approaches (iOTAs) employing telephone or digital strategies have been used to engage lower-income and underrepresented communities in lifestyle changes that promote healthy body weight. For example, the Be Fit Be Well intervention, based on the well-known Dietary Approaches to Stop Hypertension program, produced weight loss at 24 months by extending health coaching with automated telephone messaging and internet-based self-guided content to increase engagement [34]. Be Fit Be Well has become a platform for further iOTA adaptations in underrepresented groups [35-38], where access to internet and web-enabled devices may be limited. A further adaptation of Be Fit Be Well, the Working for You iOTA for lower-income hospital workers, amplified quarterly face-to-face health coach interactions with daily interactive, semiautomated short message service (SMS) text messaging [39-41]. The use of low-cost SMS technology makes the Working for You intervention an ideal iOTA for people living with SMIs, who may have limited access to smart or mobile devices or the internet [21,42,43].

In this study, we tested the usability of the Working for You iOTA in settings where patients with SMIs are most likely to engage in psychosocial rehabilitation and mental health treatment—outpatient community clinical settings [22,25]. We sought to establish criterion-level treatment engagement, defining characteristics that would allow for operationalizing engagement criteria in a scaled intervention and identifying participants less likely to engage, hypothesizing that illness severity and SMS response rate would be associated with weight change. The new operationalized inclusion criteria were then applied to evaluate whether symptom severity and early intervention engagement would impact treatment outcome. We also obtained acceptability and usability data to guide future treatment adaptation.

## Methods

### Participants

Individuals ages 16 to 75 years who were actively engaged in outpatient community mental health clinic or clubhouse programming in the St. Louis and surrounding urban, suburban, and rural areas [44-46] were eligible for participation. Clubhouses are community-based programs that provide structured daytime programming for psychosocial rehabilitation through supported educational, vocational, and social activities, referring to participants as members rather than clients or patients [47].

A priori study inclusion criteria were BMI >28 and diagnosis of a severe and persistent mental illness (recurrent major depressive disorder, schizophrenia spectrum disorder, or bipolar disorder) confirmed with medical record review. Known eating disorder diagnosis, active substance use disorder, and acute suicidality were exclusionary.

### Ethics Approval

This study was approved by the Washington University in St. Louis institutional review board (protocol number 201706118). Capacity to provide informed consent was confirmed by assessing basic medical literacy using the Rapid Estimate of Adult Literacy in Medicine–Short Form (REALM-SF), a 7-item word recognition test, with a score of 5 or higher being consistent with an 8th grade reading level and minimal ability to understand medical terminology [48] and the University of California, San Diego, Brief Assessment of Capacity to Consent (UBACC) [49], a 10-item scale that includes questions focusing on understanding of the information concerning a specific research protocol as an indicator of decisional capacity.

### Description of the Parent Intervention

The parent iOTA was developed for low-wage hospital workers participating in the Working for You study [22]. Behavioral goals, scripts for the text messages, and counseling approach in the parent iOTA Working for You were developed based on previous iterations of the intervention [39,41] and previously reported effective weight-control interventions with low-income individuals and the challenges they face in terms of behavior change [50]. Specifically, low-income individuals struggle with access to care due to transportation issues reducing engagement.

In the Working for You study, participants met one-to-one with a health coach on a quarterly basis. At the first study visit, the health coach met with participants to obtain written informed consent, review the individual’s health risk assessment, and choose up to 3 behavior change goals related to principles of energy balance. Goals were set based on behaviors identified as those (1) in highest need of change, (2) for which the participant has high self-efficacy and readiness for change, (3) for which the participant identifies few change barriers, and (4) that fulfill the intended impact on energy balance. Goals domains are based on simple, concrete behavior changes known to be effective based on empirical evidence of link to energy balance/weight and relevance to low-income populations [39,41]. Subsequent in-person health coaching visits occurred on a quarterly basis and were designed to review goal progress, problem-solve barriers to behavior change using motivational interviewing principles, and revise goal selections as needed.

Table 1 presents the options for goals from which participants could choose. Participants received text messages 5 days per week that are directly linked to goals selected in coaching sessions. Automated weekly SMS check-ins prompted a weekly reply with weight and progress toward selected goals. Participants were offered an automatic opportunity to increase the level of their selected goals if they were successful at meeting their dietary or physical activity goal target for 2 weeks in a row.

**Table 1:** Goal categories and associated health tip text messages.

Goal category		Representative text
**Activity goals**		
	Steps	It’s easy to add steps to your day! Take the stairs instead of the elevator; get off the bus a stop early.
	Brisk activity	Is your brisk activity brisk enough? A good test: You should still be able to talk easily but singing puts you out of breath.
**Dietary goals**		
	Sugar-sweetened beverages	Sugary drinks include juice, regular soda, sports/energy drinks, & sugar-sweetened teas/coffees. Cutting back is good. Zero is best.
	Healthy breakfast	A protein bar and piece of fruit can be an easy on-the-go breakfast. Choose bars with at least 10 grams of protein and 200 or fewer calories.
	Purchased meals	If you’re craving fast food, choose a lower-calorie option, like a small burger, or small fries, or small bean burrito.
	Purchased snacks	Save some money and calories by packing healthy snacks in your lunch instead of buying them at vending machines and gift shops.
	Free food	Sometimes we eat free food just because it’s free. Slow down and decide if the calories are really worth it.
	Eat meals at home	Don’t like to cook? Start simple. Try sandwiches, whole-grain cereal, canned low-sodium beans, or quick-cook brown rice. Whatever works.
	Low-fat dairy	2% milk is a good choice over whole milk. Even better is 1% or skim milk. Work your way down. You can do this!
	Fruits & Vegetables	Eating a lot of fruits & veggies can help keep hunger away and your weight in check. Work up to 5 or more servings a day.
	Vegetables	Stock up on frozen vegetables, so you can just grab them out of the freezer when you need them. Plus, they taste great and are just as healthy as fresh.
	Whole grains	Boost your whole grains with popcorn. Buy kernels and pop them in a brown paper bag in the microwave. Use a dash of powder seasoning for taste.
	High-fat meats	Beans & lentils are a great substitution for meat. You can make a lot of different dishes with them, and they’re cheap & filling.
	High-calorie snacks	Choose healthy snacks under 200 calories like 20 to 25 nuts, a banana & 1 low-fat string cheese, or baby carrots & 2 tablespoons of hummus.
	Screen time snacks	Instead of automatically grabbing a snack while watching TV, have some unsweetened tea, a diet soda, or zero-calorie fizzy water instead.
	Added calories	Tacos, burritos & nachos can be a minefield of added calories. Replace high-calorie toppings with tomatoes, salsa, lettuce, onions, and jalapenos.
	Total calories	Think of calories like money in a checking account. You can spend them any way you want, as long as they balance out at your calorie goal over time.
	Portion control	Eating slowly is a great way to feel full with smaller portions. It gives your stomach time to tell your brain when it’s had enough.
	Dietary self-monitoring	Be honest with yourself when writing down what you’re eating & drinking. Food logs help you the most when they’re as accurate as possible.

### Modifications to the Parent Intervention

Participants in this study underwent 12 weeks of treatment consisting of either monthly one-on-one in-person visits for participants seen in the outpatient clinic setting or monthly group sessions to deliver educational content, directly followed by brief one-to-one goal-setting for participants seen in the clubhouse setting. To more closely parallel the chronic care model in which most SMI patients receive care, participants in this study met monthly with health coaches. Additional treatment adaptations included adding weekly phone check-ins as needed, elements of problem-solving therapy (coaching, behavioral modeling and shaping, rehearsing and providing feedback on new behaviors, and positive reinforcement of desired health behaviors) [51] and motivational interviewing (exploring ambivalence, assessing confidence in ability to change, and shoring up self-efficacy for health behaviors) [52] in monthly in-person meetings to address barriers to behavior change. No modifications were made to the program health goals or related SMS-delivered health tips.

As in the parent intervention, participants were prompted weekly to respond via SMS text with their weight and progress toward health goals. During monthly in-person coaching meetings, the health coach reviewed progress toward goals, problem solved barriers to goal achievement, and assisted participants in selecting new goals, if necessary, at each monthly meeting based on mastery, preference, and energy balance priority. Session 1 consisted of a weigh-in and discussion of the relationship between health behaviors, health goals, and body weight (10 minutes), followed by discussion of energy balance and self-monitoring (30 minutes). The session concluded with a review of health goals, revising and scaling difficulty as appropriate (10 minutes). Subsequent visits followed the same format, with the 30-minute discussion including session-specific content (session 2: meal planning and nutrition, session 3: physical activity, and session 4: skills for long-term health behavior change). The order of the content delivered in the sessions was determined based on prior behavioral weight loss treatment development experience in youth and adults with SMI treated in community settings, where qualitative efforts identified the greatest health behavior knowledge and skill deficits in this population were understanding nutrition and healthy meal planning [46,53].

### Defining Characteristics Associated With Intervention Engagement

The primary objective of this study was to evaluate factors associated with engagement, a primary indicator of successful weight loss in behavioral lifestyle interventions [8,54]. We evaluated illness severity, treatment engagement (measured by weekly text messaging response rate), and loss-of-control eating as potential exclusion criteria. Severe psychiatric symptoms were defined as baseline clinician-administered Clinical Global Impression (CGI) [55] severity score of >5 (1=not at all ill, 2=borderline mentally ill, 3=mildly ill, 4=moderately ill, 5=markedly ill, 6=severely ill, 7=among the most extremely ill patients). Engagement was defined as text messaging response rate of >80% over the first 4 weeks of treatment.

The presence of loss-of-control eating was assessed with the 7-item Loss of Control Over Eating Score (LOCES) [56], which has demonstrated good test-retest reliability at 4 weeks (Cohen *d*=0.82, *P*<.001), strong content validity and internal consistency, factor structure, and convergent and discriminant validity [57]. The assessment was administered by a study clinician at the initial study visit. Participants were asked to rate how often they experience behavioral (“I found myself eating despite negative consequences”) and dissociative (“my eating felt like a ball rolling down a hill that just kept going and going”) symptoms associated with loss-of-control eating behavior in the prior 4 weeks (1=never, 2=rarely/once weekly, 3=sometimes/2-3 times per week, 4=often/4-5 times per week, 5=always/daily). Questions were asked in a conversational way, and feelings of shame or guardedness in responding were normalized and validated to minimize bias in reporting symptoms. Care setting (clubhouse versus community mental health clinic) was also evaluated.

### Usability, Acceptability, and Feasibility

We evaluated participant experiences with the in-person and text-messaging aspects of the intervention, specifically assessing satisfaction with treatment content, visit and text messaging frequency, and usability of the text messaging portion of the intervention. At the end of the 12-week study, participants completed a 5-question treatment satisfaction survey. Questions were based on the Contextual Technology Adaptation Process (CTAP) developed by Lyon and colleagues [58].

The CTAP model is based on user-centered design and implementation science principles and incorporates mixed quantitative and qualitative assessments considering aspects of the technology under study (eg, complexity, intended frequency of use), contexts in which the technology will be used (eg, user types and experiences, organizational setting, culture, and policies), and resources available for adaptation efforts (eg, time, money to devote to programming). CTAP involves 5 phases, including initial assessment in relevant contexts, testing of unadapted technology, adaptation, retesting, and sustained iterative assessment and adaptation processes.

This study used early-phase CTAP approaches to assess the acceptability of the unadapted technology in a new user population (adults with SMI) and treatment settings (clubhouse or community mental health clinic). Our CTAP questionnaire consisted of 5 questions focused on satisfaction and acceptability of the (1) overall program, (2) in-person health coaching visits, (3) goal options, (4) text message responses, and (5) health tip text message content. Response options were 1=very unsatisfied or unhelpful to 5=very satisfied or helpful. Each domain included 3 corresponding open-ended questions: What did you like? What did you dislike? What would you change?

### Analytic Approach

Repeated measures analysis of covariance was used to test for the effect of time on weight change, including 2-level factors for membership in included/excluded participant group (Clinical Global Impression–measured illness severity at baseline, text message response rate <80% in the first 4 weeks), and treatment setting, as well as an exploratory covariate representing score on the LOCES. Finally, we evaluated whether treatment setting for monthly in-person (one-to-one versus group) sessions influenced weight change over time. Significance was set at *P*<.05 using a 2-tailed test. CTAP responses were tabulated and presented as frequency and percentage followed by a representative quotation from corresponding open-ended questions.

## Results

### Participant Characteristics

A total of 26 participants were recruited for the study (6/26, 24% schizophrenia; 17/26, 68% mood disorder). The mean age of the overall population was 48.5 (SD 15.67) years; 60% (15/26) were white and 62% (16/26) female (Table 2). One participant was excluded per protocol for an alcohol use disorder relapse during study participation.

**Table 2:** Participant demographics and characteristics at enrollment.

Characteristic		Total (n=25)	Clubhouse (group), (n=12)	Outpatient clinic (individual), (n=13)
**Demographics**				
	Age (years), median (IQR)	50.0 (39.0-59.5)	55.0 (46.5-63.8)	48.0 (24.5-53.0)
	Male, n (%)	10.0 (40.0)	5 (41.7)	5 (38.5)
	White, n (%)	15 (60.0)	6 (50.0)	9 (69.2)
	Hispanic, n (%)	2 (8.0)	0 (0.0)	2 (15.4)
**Clinical assessments**				
	Weight (lbs), mean (SD)	224.0 (195.0-291.0)	227.0 (208.0-287.8)	220.0 (181.0-300.0)
	CGI-S^a^, median (IQR)	4.0 (3.0-4.0)	4.0 (3.3-4.0)	4.0 (3.0-4.5)
	LOCES^b^, median (IQR)	18.0 (12.0-20.0)	19.5 (18.0-28.5)	16.0 (10.5-18.5)
**Primary psychiatric diagnosis, n (%)**				
	Schizophrenia	6 (24.0)	3 (25.0)	3 (23.1)
	Bipolar disorder	12 (48.0)	8 (66.7)	4 (30.8)
	MDD^c^	5 (20.0)	1 (8.3)	4 (30.8)
	ADHD^d^	1 (4.0)	0 (0.0)	1 (7.7)
	ASD^e^	1 (4.0)	0 (0.0)	1 (7.7)

^a^CGI-S: Clinical Global Impression–Severity.

^b^LOCES: Loss of Control Over Eating Scale.

^c^MDD: Major depressive disorder.

^d^ADHD: Attention deficit-hyperactivity disorder.

^e^ASD: Autism spectrum disorder.

### Engagement Characteristics

Of the 26 participants who enrolled, 13 received in-person health coaching in a group-based clubhouse setting (mean –5.4, SD 6.9 lbs) and 12 received one-to-one health coaching with a study interventionist (mean 3.9, SD 20 lbs). One participant lost eligibility due to relapse in substance use. In the pooled treatment group, mean weight change was –0.7 (SD 15.4) lbs (min –20.5 lbs, max 58 lbs). Eight participants met the exclusion criteria under evaluation; 2 participants had a Clinical Global Impression score >5; mean weight change during treatment in this group was 32.9 (SD 25.1) lbs. Six participants had a text message response rate <80%; mean weight change in this group was 8.1 (SD 3.8) lbs.

**Figure 1:**
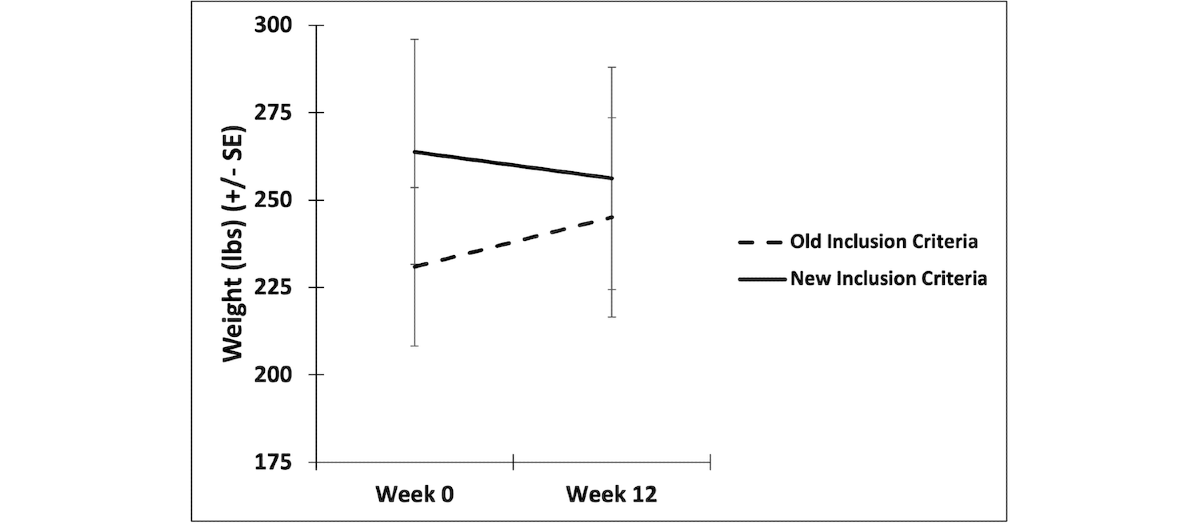
Intervention-related change in weight before and after adding engagement and illness severity inclusion criteria with standard error (SE) bars.

Using repeated measures analysis of covariance (Figure 1), a significant interaction was observed between group (eg, prior to and following application of revised inclusion criteria) and time (*F*_1,23_=17.98, *P*<.001), explained by lower symptom severity and >80% text-messaging response rate in the first 4 weeks of participation (n=18) exhibiting a significant decrease in weight (*F*_1,16_=22.54, *P*<.001). Participants with high symptom severity and low treatment engagement (n=8) had a trend-level increase in weight (*F*_1,7_=4.33, *P*=.08).

We also tested the interactive effect of time and treatment setting, and LOCES score on change in weight over 12 weeks of iOTA. No significant interactions were observed with treatment setting (*F*_1,23_=2.22, *P*=.15) or LOCES score (*F*_1,22_=0.02, *P*=.90).

### Treatment Acceptability and Satisfaction

CTAP questions regarding acceptability and satisfaction with the intervention (Table 3) and optional, open-ended questions regarding likes, dislikes, and aspects of the intervention participants would want to change (Table 4) were administered at the final study visit using a paper version of the questionnaire (Multimedia Appendix 1). Quotes were transcribed from the handwritten responses. The majority (23/26, 92%) of participants reported general satisfaction with the treatment program. Those who reported a mild level of dissatisfaction (2/26, 8%) reported that they experienced the program positively but wanted it to be longer. Participants in general (23/26, 92%) reported a moderate to high level of satisfaction with the in-person health coaching visits, with those reporting neutral or mild dissatisfaction attributing this to a desire for more phone or text message access to the health coach. In terms of in-person treatment content, most (24, 96%) participants felt the health goals offered in the unadapted program were helpful but expressed a preference for the option to personalize health goals.

The weekly SMS prompts to track weight and goal progress were viewed positively (23/26, 92%) in that they provided an easy option for accountability and self-monitoring. However, to be associated with the selected health coals, responses included prescribed tags consisting of 3-5 letters in all caps (eg, participants were reminded to type in LBS before their weekly weight) were difficult to remember; omitting the response tag or responding with an incorrect tag generated error messages that may have resulted in abandonment of the check-in process. Finally, participants in general felt the text message content was helpful but expressed a desire for customizable text messages relevant to personalized goals.

**Table 3:** Contextual Technology Adaptation Process questions.

Question	Very unsatisfied/unhelpful	Somewhat unsatisfied/unhelpful	Neutral	Somewhat satisfied/helpful	Very satisfied/helpful
How satisfied were you with the overall program?	—^a^	2 (7.7)	1 (3.8)	4 (15.4)	19 (73.1)
How satisfied were you with the amount of contact with the health coach?	—	1 (3.8)	1 (3.8)	5 (19.2)	18 (69.2)
How helpful were the health goal options offered in the program?	—	—	1 (3.8)	7 (26.9)	17 (65.4)
How helpful was the weekly weight check-in via text message?	1 (3.8)	1 (3.8)	1 (3.8)	5 (19.2)	18 (69.2)
How helpful were the daily health tip text messages?	1 (3.8)	2 (7.7)	2 (7.7)	6 (23.1)	15 (57.7)

^a^Not applicable.

**Table 4:** Representative quotes from open-ended Contextual Technology Adaptation Process questions.

Representative verbatim quotes in response to: What did you like? What did you dislike? What would you change?	Acceptability score associated with representative quote
“I’m very thankful to have been in this program. It improved my mental and physical state.”	Somewhat satisfied
“The program is too short and needs to be longer. One year would be good.”	Very satisfied
“I liked the encouragement and interaction [with] my health coach, and the helpful information I received”	Very satisfied
“Would like more calls or texts with the health coach.”	Very satisfied
“I liked the options of health goals that were offered & meeting in person—I felt motivated to make changes.”	Very satisfied
“More customizable goals.”	Very satisfied
“The accountability reminder to weigh and stay on track every week was helpful.”	Very helpful
“Responding was confusing. If I used the wrong tag to answer, I got an error message.”	Somewhat helpful
“The texts are kinda like having a little voice in the back of my head reminding me of stuff!”	Very helpful
“…More inspirational, motivational, educational texts that are longer and more frequent.”	Neutral

## Discussion

### Principal Findings

In this study, we explored the feasibility of delivering an interactive text messaging weight loss intervention to adults with SMIs treated in community clinical settings. Our initial hypothesis, driven by results from previous studies of weight loss interventions in SMI populations [16,59], was that illness severity would play an important role in engagement. Secondarily, we hypothesized that an mHealth intervention would be well accepted in this population, potentially increasing engagement and improving outcomes. We also aimed to establish minimal engagement criteria for future study by characterizing participants most and least likely to respond to this iOTA based on severity of illness [15], early engagement [60,61], and loss-of-control eating [62,63], all items that have been identified as barriers to obesity treatment success in people with SMIs. Finally, we aimed to collect usability data to identify areas for additional treatment adaptation specific to people with SMIs.

In this usability test, engagement, measured by percentage response to SMS messages within the first month of participation, and lower severity of illness at baseline predicted change in weight at 12 weeks. Qualitative data on user experience and user satisfaction indicated that participants felt positively toward text messaging. Participants still felt that in-person visits were important but would also be acceptable if done remotely via telehealth or text. Like prior studies testing usability of health behavior change apps [64], participants with SMIs wanted more personalized goals and texts and simplified ways to respond to accountability check-ins. Longer term engagement was also preferred by many and is consistent with recommended length of treatment [31,65].

### Comparison With Prior Work

It is well known that obesity and mental illness are highly comorbid, with most patients seeking obesity treatment having two or more treatable diagnoses [11,12]. Further, individuals who receive appropriate treatment to stabilize psychiatric symptoms experience benefits of obesity treatments and have similar outcomes to those without psychiatric illnesses [66]. However, clinical populations with mental health conditions have much higher rates of obesity [67,68] and increased risk for cardiometabolic conditions like diabetes and hypertension than the general population [69-71]. They also exhibit attenuated response to unadapted obesity treatments, which is thought to be related to numerous hampering factors that decrease engagement, including low motivation and cognitive difficulties [22,53], socioeconomic disadvantages such as limited transportation [72], and psychosocial disadvantages preventing engagement in clinical services that could be reduced with mHealth approaches [73]. People with SMIs are amenable to mHealth interventions for weight management [74] and already have access to and familiarity with low-tech methods like SMS [21]. Combined interventions that include in-person visits with mHealth extenders for coaching might be reasonably implemented in settings where monthly or more frequent in-person visits are part of the existing care structure.

### Limitations

This study is subject to important limitations; namely, the sample size is small and relatively heterogeneous in terms of diagnosis, care setting, and baseline weight and age. Thus, adequate power to detect between-group differences in subgroup analyses is limited. It should also be noted that the inclusion of patients engaged in services only limits the generalizability of the results; people with SMI are often disengaged from clinical care for a variety of reasons having to do with disparity and disadvantage [75]. As a result, individuals who are not engaged in care may have needs and preferences that could be uniquely addressed by a digital health intervention [76] but who are not reached by recruiting only from populations encountered in clinical care settings. Additionally, while treatment content and procedures were consistent regardless of treatment setting, feasibility of implementation in each setting was not formally assessed. Further study is needed to evaluate additional adaptation needs specific to treatment setting, including qualitative assessment of implementation barriers and facilitators from perspectives of decision-makers, clinicians, and patients.

### Conclusions

These data are relevant to future study design considerations and support further testing of specific exclusion criteria for defining treatment engagement and psychiatric symptom control. Future studies applying these selection criteria may be important in evaluating the effect of this iOTA in SMI but will need to consider methods for reaching individuals who may benefit most from mHealth interventions—those who are disengaged from care, from underrepresented or disadvantaged populations, or who live in rural areas. Finally, more comprehensive symptom assessments may be needed to understand the effect of eating disorder symptoms on weight change outcomes in this populations. Nonetheless, these results demonstrate the feasibility of delivering an adapted iOTA intervention to SMI patients receiving care in clubhouse and community mental health clinic settings and suggest testable criteria for defining sufficient treatment engagement and psychiatric symptom severity.

## Appendix

Multimedia Appendix 1 Behavioral assessment questionnaire.

## Abbreviations

CTAP: Contextual Technology Adaptation Process
iOTA: interactive obesity treatment approach
LOCES: Loss of Control Over Eating Scale
mHealth: mobile health
NIH: National Institutes of Health
SMI: severe mental illness
SMS: short message service
WUSM: Washington University School of Medicine
