# Supplementary material for: Use of an Interactive Obesity Treatment Approach in Individuals With Severe Mental Illness: Feasibility, Acceptability, and Proposed Engagement Criteria
Source: JMIR Form Res. 2022 Dec 13;6(12):e38496. doi: 10.2196/38496 (PMC9795399; doi:10.2196/38496)
Supplement: Multimedia Appendix 1 [file formative_v6i12e38496_app1.pdf]

Interactive Obesity Treatment Approach
in individuals with severe mental illnesses (iOTA in SMI)

Participant ID: ____________
Visit #: ____ Date: ________

Rapid Estimate of Adult Literacy in Medicine Short Form (REALM-SF)

Instructions:

1. Give the participant a paper copy of the REALM-SF form and score answers on a separate paper copy that is attached to a clipboard. Hold the clipboard at an angle so that the participant is not distracted by your scoring.

   Say:
   "I want to hear you read as many words as you can from this list. Begin with the first work and read aloud. When you come to a word you cannot read, do the best you can or say, 'blank' and go on to the next word."

2. If the patient takes more than 5 seconds on a word, say 'blank' and point to the next word, if necessary, to move the patient along. If the patient begins to miss every word, have him or her pronounce only known words.

Antibiotics	☐
Behavior	☐
Anemia	☐
Rectal	☐
Jaundice	☐
Exercise	☐
Menopause	☐

Scoring and Grade Equivalents:

Score	Grade Range
0	Third grade and below; will not be able to read most low-literacy materials; will need repeated oral instructions, materials composed primarily of illustrations, or audio or video tapes.
1 – 3	Fourth to sixth grade; will need low-literacy materials, may not be able to read prescription labels.
4 – 6	Seventh to eighth grade; will struggle with most patient education materials; will not be offended by low-literacy materials.
7	High school; will be able to read most patient education materials.

Adapted from: Arozullah, A.M., Yarnold, P.R., Bennett, C.L., Soltysik, R.C., Wolf, M.S., Ferreira, R.M., Lee, S.Y., Costello, S., Shakir, A., Denwood, C., Bryant, F.B., & Davis, T. (2007). Development and validation of a short-form, rapid estimate of adult literacy in medicine. Med Care, 45(11):1026-33.

University of California, San Diego Brief Assessment of Capacity to Consent (UBACC)

Instructions: After reviewing study details and the informed consent document, explain that you are going to ask a few brief questions about the study. Participants should be allowed to refer to the Informed Consent Form when answering these questions but should be encouraged to respond in their own words. If a participant has trouble understanding one of the questions on the UBACC, rephrase the question. Rate the participant's responses on a scale of 0 – 2, with "0" being the lowest (little to no understanding of this aspect of the study) and "2" being the highest (clear understanding of this aspect of the study).

A score of 15 or higher is needed for inclusion in the study. If a patient scores lower than 15, the person obtaining consent can review the study details and have the patient return on another day to re-do the UBACC and obtain consent.

Question	Understanding Score		
	0	1	2
	Poor	Some	Clear
**What is the purpose of the study that was just described to you?**<br>(2 = compare weight loss between two different text-messaging interventions)			
**What makes you want to consider participating in this study?**<br>(2 = Learn healthy habits, lose weight)			
**Do you believe this is primarily research or primarily treatment?**<br>(2 = Research)			
**Do you have to be in this study if you don't want to participate?**<br>(2 = No)			
**If you withdraw from this study, will you still be able to receive regular treatment?**<br>(2 = Yes)			
**If you participate in this study, what will you be asked to do?**<br>(2 = Attend monthly visits, respond to text messages)			
**Describe some risks or discomforts that may occur if you participate in this study.**<br>(2 = I might not lose weight; text messages might be annoying/hard to understand)			
**Please describe some of the possible benefits of this study.**<br>(2 = I might learn healthy lifestyle skills, I may lose weight)			
**Is it possible that being in this study will not have any benefit to you?**<br>(2 = Yes)			
**Who will pay for medical care if you're injured due to participating in this study?**<br>(2 = These costs would be billed to me or my insurance company)			

Total Score (sum) ___________

Adapted from: Jeste DV, Palmer BW, Appelbaum PS, Golshan S, Glorioso D, Dunn LB, Kim K, Meeks T, Kraemer HC. A New Brief Instrument for Assessing Decisional Capacity for Clinical Research. Arch Gen Psych 2007;64(8):966-974

Participant ID: ____________
Visit #: ____ Date: ________

7-item Loss of Control Over Eating Scale-Brief (LOCES-Brief)

Instructions: Select how often the participant has experienced the following while eating in the last 4 weeks (28 days). The final score is the average of the sum.

Never	Once a month or less
Rarely	Once weekly
Sometimes	2-3 times per week
Often	4-5 times per week
Always	Daily or more

Frequency	Never	Rarely	Some-times	Often	Always
**Score**	**1**	**2**	**3**	**4**	**5**
I continued to eat past the point when I wanted to stop.					
I felt like I had "blown it" and might as well keep eating.					
I felt helpless about controlling my eating.					
My eating felt like a ball rolling downhill that kept going & going.					
I found myself eating despite negative consequences.					
I felt like the craving to eat overpowered me.					
I felt like I could not do anything other than eat.					

Sum Total: _________

Average: _________

Adapted from: Latner, JD; Mond, JM; Kelly, MC; Haynes, SN; Hay, PJ. The Loss of Control Over Eating Scale: development and psychometric evaluation. Int J Eat Disord. 2014 Sep; 47(6):647-59.

Interactive Obesity Treatment Approach
in individuals with severe mental illnesses (iOTA in SMI)

Participant ID: ____________
Visit #: ____ Date: ________

Treatment Satisfaction & Acceptability:
Contextual Technology Adaptation Process (CTAP) Questionnaire

Instructions: For the numbered questions in **BOLD** font, select the box that fits your answer. If applicable, you can write out an answer for the three questions in *itallics* underneath each **BOLD** question.

	Very Unsatisfied	Somewhat Unsatisfied	Equally Unsatisfied and Satisfied	Somewhat Satisfied	Very Satisfied
**1. How satisfied are you with the overall program?**	☐	☐	☐	☐	☐
What did you like?					
What did you dislike?					
What would you change?					
	**Very Unsatisfied**	**Somewhat Unsatisfied**	**Equally Unsatisfied and Satisfied**	**Somewhat Satisfied**	**Very Satisfied**
**2. How satisfied are you with the in-person visits with your health coach?**	☐	☐	☐	☐	☐
What did you like?					
What did you dislike?					
What would you change?					

	Very Unhelpful	Somewhat Unhelpful	Equally Helpful and Unhelpful	Somewhat Helpful	Very Helpful
**3. How helpful were the health goal options offered in the program?**	☐	☐	☐	☐	☐
What did you like?					
What did you dislike?					
What would you change?					
	**Very Unhelpful**	**Somewhat Unhelpful**	**Equally Helpful and Unhelpful**	**Somewhat Helpful**	**Very Helpful**
**4. How helpful was the weekly weight check-in by text message?**	☐	☐	☐	☐	☐
What did you like?					
What did you dislike?					
What would you change?					
	**Very Unhelpful**	**Somewhat Unhelpful**	**Equally Helpful and Unhelpful**	**Somewhat Helpful**	**Very Helpful**
**5. How helpful were the health tips you received by text message?**	☐	☐	☐	☐	☐
What did you like?					
What did you dislike?					
What would you change?					

Based on the Contextual Technology Adaptation Process (CTAP) outlined by Lyon and colleagues:

Lyon AR, Wasse JK, Ludwig K, Zachry M, Bruns EJ, Unutzer J, et al. The Contextualized Technology Adaptation Process (CTAP): Optimizing Health Information Technology to Improve Mental Health Systems. Adm Policy Ment Health. 2016;43(3):394-409.
